# Hypertensive Choroidopathy With Bilateral Serous Retinal Detachment as the Presenting Finding in Malignant Hypertension

**DOI:** 10.1155/crop/2928187

**Published:** 2025-10-09

**Authors:** Ali Kutay Kılınç, Ali Aydın, Cahit Burke

**Affiliations:** Department of Ophthalmology of Near East University Hospital, Lefkoşa, Cyprus

**Keywords:** hypertensive choroidopathy, malignant hypertension, secondary hypertension, serous macular detachment

## Abstract

**Background:**

Secondary hypertension, particularly in young patients, can manifest as malignant hypertension with ocular complications. We report a case of hypertensive choroidopathy with serous retinal detachment as the initial presentation of malignant hypertension in a young woman with renal failure secondary to antimalarial treatment.

**Case Presentation:**

A 19-year-old woman with a history of renal failure following antimalarial treatment presented with sudden, bilateral vision loss (right eye: 20/200, left eye: 20/40). Fundus examination revealed asymmetrical choroidal involvement with serous retinal detachment, more pronounced in the right eye. Her blood pressure was markedly elevated at 230/130 mmHg. Following blood pressure control, the patient's vision improved to 20/20 in both eyes and the serous retinal detachments resolved completely.

**Conclusion:**

This case highlights the importance of considering hypertensive choroidopathy in young patients with secondary hypertension presenting with sudden vision loss. Prompt diagnosis and blood pressure management can lead to complete visual recovery.

## 1. Introduction

Malignant hypertensive crisis (MHT) is a cardiovascular emergency in which arterial blood pressure increases to a level that can lead to organ damage [1]. Guidelines on hypertension describe MHT or emergency as systolic blood pressure ≥ 180 mmHg and/or diastolic blood pressure ≥ 120 mmHg with organ damage [2]. Extremely high hypertension can cause damage to organs such as the eyes, kidneys, and brain through microvascular circulation impairment. If left untreated, MHT can cause angiopathy in the fundus, leading to optic neuropathy, retinopathy, and/or choroidopathy. Hypertensive choroidopathy (HTC) is much less common than other fundus complications and is usually seen in young patients due to the elasticity of blood vessels with contractile function [3]. Besides, ophthalmological complaints may be the first manifestation of hypertension, especially in young people [4, 5]. Herein, we present a young female patient who presented with a sudden decrease in vision due to the HTC with serous retinal detachment as the first finding of MHT.

## 2. Case Presentation

A 19-year-old African female presented to the Ophthalmology Department of Near East University Hospital, Lefkoşa, Cyprus, with a 2-day history of blurred vision. Her systemic medical history included a diagnosis of malaria 2 months prior, for which she received medication in Nigeria before arriving in Cyprus. During her current admission and following ophthalmic examination, she was diagnosed with malignant hypertension and acute renal failure, which were identified as a complication of the antimalarial therapy. This renal condition later necessitated a kidney transplant within a year.

**Figure 1:**
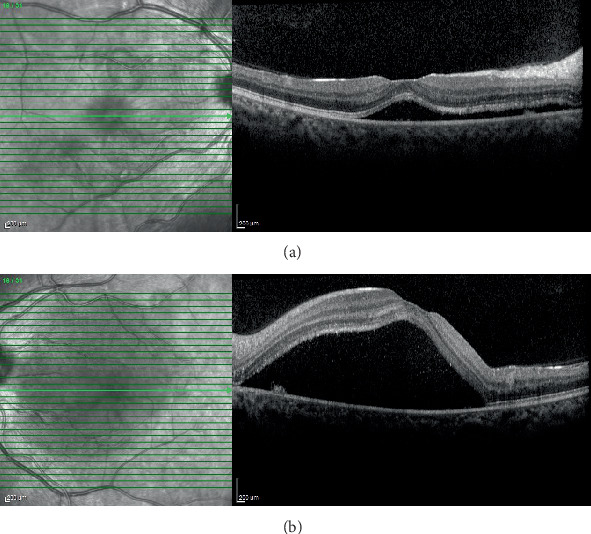
(a, b) SD-OCT. Central subretinal fluid with neurosensory detachment, subretinal hyperreflective dots, and accumulation over retinal pigment epithelium.

**Figure 2:**
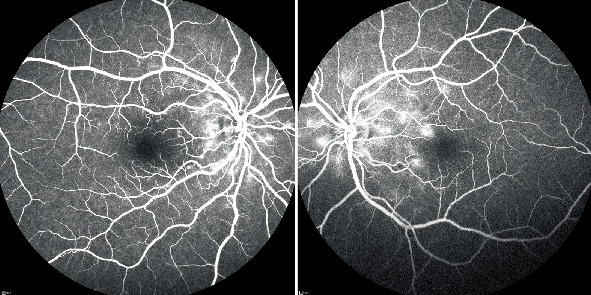
Fundus fluorescein angiography. Late arteriovenous phase angiogram (arm–retina time right eye 01:53, left eye 01:57) of the right and left eyes. Multiple patchy hyperfluorescent and leaking spots with FFA showed describing Elschnig spots. Areas of capillary nonperfusion can be recognised as dark areas lacking the dye.

On ophthalmic examination, best-corrected visual acuity was 20/200 in the right and 20/40 in the left eye with a myopic correction of −1.75 diopter (D) in both eyes. Slit lamp examination and pupillary reactions were within normal limits. Intraocular pressure was 12 mmHg in both eyes. Fundus examination revealed no pathology such as vitritis, choroiditis lesions, optic disc swelling, retinal hemorrhage, or exudate, except macular elevation in both eyes. Macular elevation was more noticeable in the right eye. Spectral domain optical coherence tomography (Spectralis, Heidelberg Engineering Inc., Heidelberg, Germany) showed submacular fluid accumulation consistent with serous retinal detachment in both eyes without significant thickening of the choroidal layer (Figure 1). Fundus fluorescein angiography revealed multiple patchy hyperfluorescent and leaking spots, presumed to be choroidal in origin; however, definitive confirmation via Indocyanine Green Angiography was not possible in our clinic due to its absence (Spectralis HRA 2, Heidelberg Engineering Inc., Heidelberg, Germany) (Figure 2).

Our diagnostic approach initially focused on excluding alternative etiologies for the patient's macular neurosensory detachment. The patient had no history of ocular trauma. Considering the patient's age, conditions such as macular degeneration or polypoidal choroidal vasculopathy were deemed improbable. Ocular measurements and funduscopic findings did not indicate degenerative myopia. In the systemic evaluation, the patient was reported to be experiencing a nonspecific, dizziness-like condition that she could not clearly define. Differential diagnoses considered included bilateral conditions such as central serous chorioretinopathy, Vogt–Koyanagi–Harada disease, hematological disorders such as leukemia or lymphoma, HTC, and drug-induced choroidopathy. Based on the absence of Elschnig spots and Siegrist lines (typical findings in HTC) and hypertensive retinopathy on fundus examination, preliminary diagnoses included Vogt–Koyanagi–Harada disease or hematological pathologies. However, we confirmed the diagnosis of MHT with a quick and practical blood pressure measurement. Consequently, the patient was urgently referred to the emergency department for treatment and investigation of the etiology of hypertension. Diagnostic tests indicated acute renal failure (creatinine 3.2 mg/dL and no postrenal obstruction on ultrasonography), leading to the patient's admission to the nephrology clinic. Magnetic resonance imaging of the brain and orbits was performed to rule out other etiologies of acute vision loss and assess for any neurological sequelae of the hypertensive crisis, showing no evidence of acute infarction or other intracranial pathologies. The absence of aberrant hematological parameters and the significant improvement in visual acuity during subsequent examinations served to validate this diagnosis.

**Figure 3:**
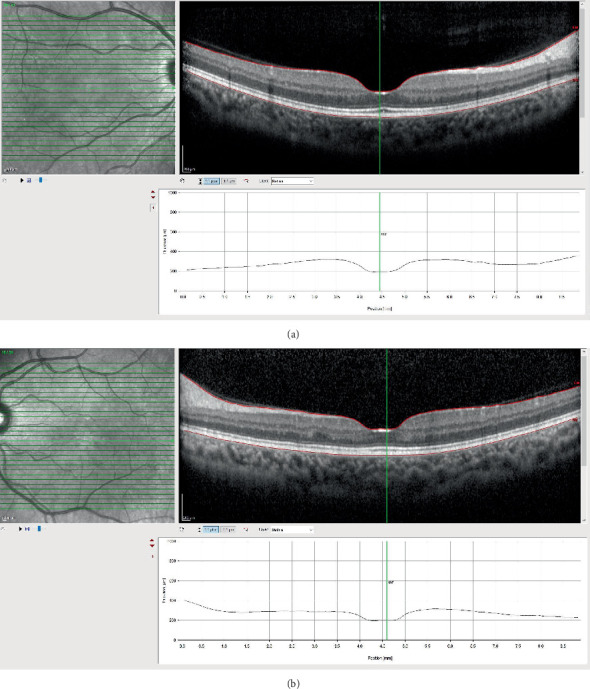
(a, b) SD-OCT. Subretinal fluid disappeared in both eyes and the foveal contour appeared natural. Irregularity in the outer plexiform layer in the left eye and hyperreflectivity in the foveal pit in both eyes are noted.

The patient's secondary hypertension was brought under control, but since the renal failure could not be treated medically, the patient was started on hemodialysis. The patient's blurred vision complaint regressed within days when examined at the bedside. No additional pathology was observed during bedside examinations. Upon her first postdischarge follow-up visit, conducted 1 month after the initial assessment, her best-corrected visual acuity in both eyes improved to 20/20. Macular OCT test showed complete resolution of the serous retinal detachment. A distinct, brighter than usual reflection was observed on the internal limiting membrane (Figure 3a,b). Notably, the patient did not receive corticosteroids during hospitalization. One year later, we learned that the patient had undergone a kidney transplant in her country.

## 3. Discussion

The worldwide prevalence of hypertension in 2010 was estimated to be 1.39 billion people, representing 31% of all adults [6]. Hypertension can primarily affect the eye by causing retinopathy (atherosclerosis of retinal vessels and vascular hyalinization in the internal blood–retinal barrier), choroidopathy (fibrinoid necrosis of choriocapillaris arterioles), and optic neuropathy [7]. HTC is thought to be more common in younger patients due to the elasticity of their blood vessels. In response to acute systemic hypertension, the flexible choroidal arterioles undergo constriction, causing a lack of perfusion of the choriocapillaris [8]. Endothelial damage leads to fibrinoid necrosis of the choroidal arterioles and choriocapillaris occlusion, resulting in focal ischemic damage to the retinal pigment epithelium (RPE). The pumping capacity of the RPE is also affected. This results in the breakdown of the outer blood–retinal barrier, so serous retinal detachments may develop. The flow defects observed in the choriocapillaris in optical coherence tomography angiography studies of serous retinal detachment with HTC confirmed the hypothesis that ischemia is an underlying mechanism of HTC [9].

Similar to our patient (Figure S4a and S4b), the patient described in the case report presented by Hirano et al. had no significant fundoscopic findings other than macular detachment. However, unlike our patient, this patient was 50 years old and had cystoid macular edema accompanying macular detachment, and no active leakage was observed on angiography. Nevertheless, they confirmed choroidal hypoperfusion with indocyanine angiography and demonstrated that HTC cannot be ruled out even if there is no leakage on fundus angiography [10]. Our patient had a hypertensive crisis at a young age and choroidal involvement secondary to renal failure. The choroidal circulation may be affected earlier than the retinal circulation by rapidly rising blood pressure, possibly due to anatomical differences and autoregulatory mechanisms in the retinal vasculature. While many studies in the literature describe sudden vision loss as the first symptom of preeclampsia or HELLP syndrome, our study observed acute renal failure and secondary hypertension presenting with vision loss as the initial symptom [11, 12, 13]. In conclusion, in young patients like ours, a hypertensive crisis may lead to choroidopathy and serous retinal detachment as the initial ocular manifestations, with resultant vision decrease serving as the first sign of the crisis. Therefore, a hypertensive crisis should also be considered in the differential diagnosis of young patients presenting with a sudden decrease in vision associated with serous retinal detachment.

Acknowledging the detrimental effects of chloroquine on the retinochoroidal microvasculature and RPE, the patient's attainment of normal visual acuity following serous macular detachment emphasizes the critical significance of preserving the outer retinal layers and the absence of retinopathy [14]. Further research is needed to evaluate the efficacy and role of potential neuroprotective antihypertensive agents in reducing blood pressure and the regression of chorioretinopathy. The resolution of serous retinal detachment is primarily dictated by management of the underlying pathology. Observation may be beneficial in the presence of neurosensory macular detachment. However, in scenarios where spontaneous resolution does not occur, it is essential to consider alternative pathophysiological mechanisms and undertake additional investigations and treatments.

## Data Availability

The data collected for this study cannot be publicly shared in order to protect the privacy and confidentiality of the participant. The datasets for the analysis of the current study are reserved by the author. Datasets are available from the corresponding author on reasonable requests.

## Nomenclature

MHT: malignant hypertensive crisis
HTC: hypertensive choroidopathy

## Supporting Information

Supporting Information Additional supporting information can be found online in the Supporting Information section. Figure S4 (a) Right eye. Infrared fundus photography of the patient (serous retinal detachment, same time with Figure 2). (b) Left eye. Infrared fundus photography of the patient (serous retinal detachment, same time with Figure 2).
