# Supplementary figures and images for: Hypertensive Choroidopathy With Bilateral Serous Retinal Detachment as the Presenting Finding in Malignant Hypertension
Source: Case Rep Ophthalmol Med. 2025 Oct 9;2025:2928187. doi: 10.1155/crop/2928187 (PMC12530919; doi:10.1155/crop/2928187)

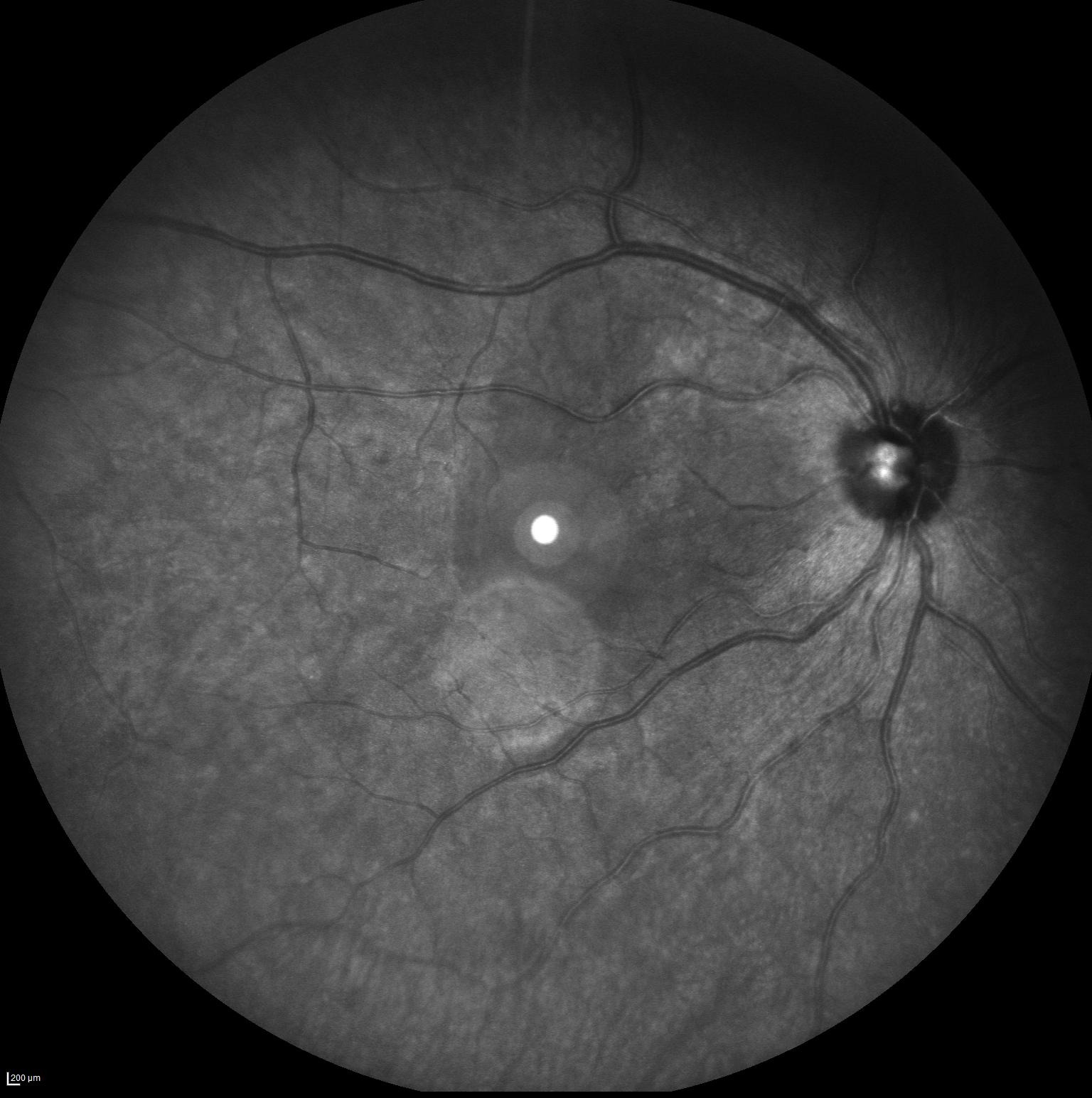

Supplement: Supporting Information — Additional supporting information can be found online in the Supporting Information section. Figure S4 (a) Right eye. Infrared fundus photography of the patient (serous retinal detachment, same time with Figure 2). (b) Left eye. Infrared fundus photography of the patient (serous retinal detachment, same time with Figure 2). [file 2928187.f1.zip › figure 4a.JPG]

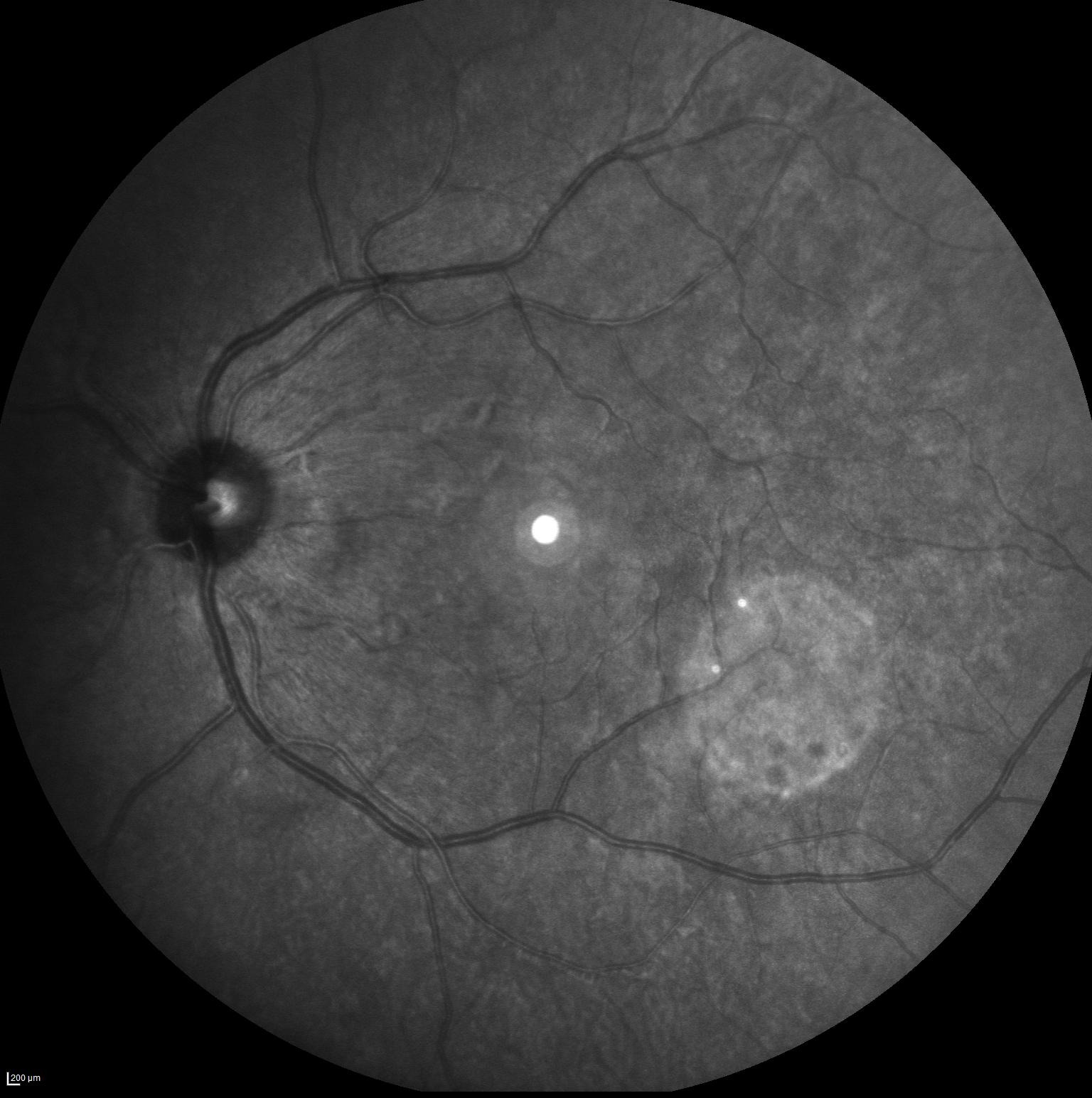

Supplement: Supporting Information — Additional supporting information can be found online in the Supporting Information section. Figure S4 (a) Right eye. Infrared fundus photography of the patient (serous retinal detachment, same time with Figure 2). (b) Left eye. Infrared fundus photography of the patient (serous retinal detachment, same time with Figure 2). [file 2928187.f1.zip › figure4b.JPG]
